# Geometric algebra based recurrent neural network for multi-dimensional time-series prediction

**DOI:** 10.3389/fncom.2022.1078150

**Published:** 2022-12-22

**Authors:** Yanping Li, Yi Wang, Yue Wang, Chunhua Qian, Rui Wang

**Affiliations:** ^1^School of Communication and Information Engineering, Shanghai University, Shanghai, China; ^2^Office of Academic Affairs, Shanghai University, Shanghai, China; ^3^Department of Endocrinology and Metabolism, Shanghai Tenth People's Hospital, School of Medicine, Tongji University, Shanghai, China

**Keywords:** geometric algebra, recurrent neural network, long-and short-term time-series network, prediction, multi-dimensional time-series

## Abstract

Recent RNN models deal with various dimensions of MTS as independent channels, which may lead to the loss of dependencies between different dimensions or the loss of associated information between each dimension and the global. To process MTS in a holistic way without losing the inter-relationship among dimensions, this paper proposes a novel Long-and Short-term Time-series network based on geometric algebra (GA), dubbed GA-LSTNet. Specifically, taking advantage of GA, multi-dimensional data at each time point of MTS is represented as GA multi-vectors to capture the inherent structures and preserve the correlation of those dimensions. In particular, traditional real-valued RNN, real-valued LSTM, and the back-propagation through time are extended to the GA domain. We evaluate the performance of the proposed GA-LSTNet model in prediction tasks on four well-known MTS datasets, and compared the prediction performance with other six methods. The experimental results indicate that our GA-LSTNet model outperforms traditional real-valued LSTNet with higher prediction accuracy, providing a more accurate solution for the existing shortcomings of MTS prediction models.

## 1. Introduction

Multi-dimensional time-series (MTS) are ubiquitous in our daily lives, including stock market prices, traffic flow on highways, output from solar power plants, temperatures in different cities, and so on. Prediction of these time-series can serve as the basis for many practical applications. However, there are usually complex dynamic interdependencies among the variables of these data (Faloutsos et al., 2018), and how to capture and utilize this information for efficient and reliable prediction is a long-standing research hotspot.

Traditional methods for MTS prediction include linear support vector regression (Cao and Tay, 2003), autoregressive integrated moving average models (Gurland and Whittle, 1951), vector autoregression (VAR) (Han et al., 2015), and so on. The most common approach is to treat observations at a point in time as vectors and model dynamic time using VARs and linear dynamical systems. Essentially, these models rely on AR coefficient matrices or dynamical matrices to capture the correlation structure between different time-series. Chen et al. (2021) extended the VAR model for third-order tensor time-series by introducing two AR coefficient matrices to characterize the correlation structure. Mohammad et al. (2019) also achieved excellent results in time-series data prediction using a stochastic model approach. However, the large number of parameters in the coefficient matrix and the high computational cost make these model parameters very difficult to estimate and prone to overfitting. At the same time, the performance of these traditional models in time-series with mixed long-and short-term modes is always insufficient, and cannot accurately capture the complex nonlinear relationship between sequence data.

Due to the excellent performance of deep learning in applications such as image recognition and machine translation, its potential in the field of MTS prediction has also attracted a lot of attention. Recent studies have indicated that modern deep learning techniques not only achieve state-of-the-art prediction performance but also systematically reduce the complexity of the prediction process significantly, thus improving maintainability (Hochreiter and Schmidhuber, 1997).

Recurrent neural network (RNN) and long short-term memory (LSTM) (Hochreiter and Schmidhuber, 1997) are the earliest neural networks to deal with time-series. Subsequently, the emergence of gated recurrent units (Cho et al., 2014) reduces the number of network parameters, reducing the risk of overfitting compared to LSTMs. At the same time, there are also many works showing that specific convolutional neural network structures can also achieve good results, such as convolution-based gated linear units (Dauphin et al., 2017) and temporal convolutional networks (Bai et al., 2018). Due to the complex structural information among the dimensions of multi-dimensional time-series, it is difficult for a single network to achieve a good processing effect. Therefore, the emergence of hybrid deep networks has brought prediction accuracy to a new level. LSTNet (Ai et al., 2017) combines CNN, LSTM, attention mechanism, and AR autoregressive process to extract short-term local dependence patterns among variables and long-term dependence patterns of time-series. DeepState (Syama et al., 2018) combines state-space models with deep recurrent neural networks to learn the parameters of the whole network by maximum log-likelihood function. DeepGLO (Sen et al., 2019) is a hybrid model that includes a global matrix decomposition model normalized by a temporal convolutional network and a temporal network that captures the local properties of each time-series and associated covariates.

In addition, graph convolutional neural networks (GCNs) have also been shown to capture the correlation between partial time-series. Spatio-temporal graph convolutional neural network (ST-GCN) (Yu et al., 2018) is a deep learning framework for traffic prediction, which fully exploits the graph structure of road networks by integrating graph convolutions and gated linear units for faster training. Li et al. (2017) directly stacked graph convolution and temporal modules to capture spatial and temporal dependencies in traffic data streams, but the network requires predefined relational topology. Graph wavenet (Wu et al., 2019) combines graph convolution layers, adaptive adjacency matrices, and expanded stochastic convolution to capture spatio-temporal dependencies. However, most of them either ignore the correlation between data or require reliance on the graph as a priori. In addition, the Fourier transform has shown its advantages in previous work, especially the joint Fourier transform (Grassi et al., 2018; Isufi et al., 2019; Loukas and Perraudin, 2019), which enables prediction tasks in the fields of weather information, traffic data, and seismic waveforms. The discrete Fourier transform can also be used for time-series analysis. For example, state-frequency memory networks (Zhang et al., 2017) combine the advantages of the discrete Fourier transform and LSTM for stock price prediction together. Nevertheless, none of the existing solutions jointly captures temporal patterns and multivariate correlations in the spectral (Parcollet et al., 2019).

Accurate prediction based on historical time-series data is challenging because it requires joint modeling of the temporal patterns of the data and correlations among the data. How to capture and exploit the dynamic correlations among multiple variables is a huge research challenge for MTS prediction. At present, there are more studies based on multi-dimensional time-series prediction, however, they are all based on the real number, and there is no more advanced theoretical breakthrough. Moreover, in the process of multi-dimensional time-series processing and final fusion, real-valued networks have the problem of information loss inevitably. It is worth noting that Parcollet et al. (2019) constructed new quaternion recurrent neural networks and quaternion long short-term memory networks with quaternions, placing the three feature values of speech signals on each of the three imaginary parts of the quaternion, and exploiting the potential structural dependence within the quaternion to obtain better performance than real RNNs and LSTMs in practical automatic speech recognition applications. However, for signals with more features, such as MTS that usually have dozens or even hundreds of features, quaternion recurrent neural networks and quaternion long short-term memory networks are powerless.

Geometric algebra (GA) has opened up new directions for the study and application of MTS. Through the potential structural dependence within the multi-vector, the multi-dimensional features are combined into a single entity input to the network for processing, capturing the internal relationships between the sequence features, allowing the structural information inherent in the multi-dimensional features to be well preserved.

Therefore, this paper proposes new geometric algebra based recurrent neural network (GA-RNN) and geometric algebra based long-and short-term time-series network (GA-LSTM) and constructs a new geometric algebra based long-and short-term time-series network, (GA-LSTNet). Firstly, the multi-dimensional time-series is represented as a GA multi-vector, preserving the correlation states of its channels. Secondly, each layer of the network and the training algorithm are extended to the GA space, and the corresponding processing algorithm is provided for the input GA multi-vector to ensure the retention of multi-channel information in signal processing. Essentially, each feature of the multi-dimensional signal is mapped to each component of the GA multi-vector separately, and then the overall computation based on the GA multi-vector is performed to maximize the retention of the potential features of the multi-dimensional signal.

The rest of this paper is organized as follows. Section 2 introduces the basics of GA and neural networks based on GA. Section 3 describes the proposed GA-RNN and GA-LSTM. Comparison experimental results between GA-LSTNet and real-valued methods are provided in Section 4, followed by concluding remarks drawn in Section 5.

## 2. Preliminary

GA was first described by William K. Clifford, also called Clifford Algebra. For multi-dimensional signals, GA is not only an effective framework to handle the representation and computation issues but also a useful tool for widespread use in mathematics and physics (Hestenes, 1986; Rafal, 2004; López-González et al., 2016).

Mathematically, suppose *G*_*n*_ denotes a 2^*n*^ dimensional vector space, and there exist a set of orthogonal bases {*e*_1_, *e*_2_, …, *e*_*n*_}. The power set γ = {1, ⋯ , *n*} can turn the basis into an ordered one with the index set Γ.


(1) $$\begin{array}{l} \Gamma :=\left\{\left(a_{1},\cdots a_{r}\right)\in \gamma ,1\leq a_{1}\cdots a_{r}\leq n\right\} \end{array}$$

Then, the basis of *G*_*n*_ is denoted by


(2) $$\begin{array}{l} \left\{e_{I}:=e_{a_{1}}\cdots e_{a_{r}}|I\in \Gamma \right\} \end{array}$$

For example, the basis in the 2^3^ vector space can be described as


(3) $$\begin{array}{l} \left\{1,e_{1},e_{2},e_{3},e_{12},e_{13},e_{23},e_{123}\right\}. \end{array}$$

For convenience, in the rest of the paper, *e*_1_⋯*e*_*r*_ will be denoted by *e*_1⋯*r*_. In general, the multiplication in GA will follow the following rules


(4) $$\{\begin{array}{l} e_{i}^{2}=1,\;i=1,\ldots ,p\\ e_{i}^{2}=-1,\;i=p+1,\ldots ,p+q \end{array}$$

and *G*_*n*_ can also be denoted as *G*_*p, q*_, with 2^*n*^ = *p*+*q*. An arbitrary element of the GA is given by


(5) $$\begin{array}{l} x=\overset{n}{\underset{t=0}{\sum }}{\langle x\rangle }_{t}=\underset{I\in \Gamma }{\sum }{\left[x\right]}_{I}e_{I} \end{array}$$

Where [*x*]_*A*_∈ℝ, represents the value of each component of the multi-vector. For example, an element in the 2^3^ vector space can be represented as


(6) $$\begin{array}{l} \begin{array}{c} x={\langle x\rangle }_{0}+{\langle x\rangle }_{1}+{\langle x\rangle }_{2}+{\langle x\rangle }_{3}\\ =x_{0}+x_{1}e_{1}+x_{2}e_{2}+x_{3}e_{3}\\ +x_{12}e_{12}+x_{13}e_{13}+x_{23}e_{23}+x_{123}e_{123} \end{array} \end{array}$$

The addition in GA can be defined as


(7) $$\begin{array}{l} x+y=\underset{I\in \Gamma }{\sum }\left({\left[x\right]}_{I}+{\left[y\right]}_{I}\right)e_{I} \end{array}$$

The geometric product in GA can be written in the following form


(8) $$\begin{array}{l} x{⊗}_{p,q}y=x\cdot y+x\wedge y \end{array}$$

Where *x*·*y* and *x*∧*y* represent the inner and outer products in GA, respectively.

The geometric product between two multi-vectors can also be converted into matrix operations. Assuming that there is a multi-vector, the multi-vector can be expressed as


(9) $$\begin{array}{l} \begin{array}{c} x=\left[{\left[x\right]}_{0},{\left[x\right]}_{1},{\left[x\right]}_{2},\ldots ,{\left[x\right]}_{I},\ldots \right]\cdot {\left[1,e_{1},e_{2},\ldots ,e_{I},\ldots \right]}^{T}\\ =F_{x}\cdot N_{x} \end{array} \end{array}$$

Where $F_{x}\in {\mathbb{R}}^{1\times 2^{n}}$ is the coefficient matrix of multi-vector *x* and *N*_*x*_ is the corresponding orthogonal basis matrix. According to the calculation rules between different *e*_*I*_, ***R***(*x*) can be defined as its real representation matrix (Roy et al., 2020). Then,


(10) $$\begin{array}{l} x{⊗}_{p,q}y=\left[R\left(y\right)\cdot {\left(F_{x}\right)}^{T}\right]\cdot N_{x\left(y\right)} \end{array}$$

The inversion of multi-vector is denoted by


(11) $$\begin{array}{l} \tilde{x}=\overset{n}{\underset{t=0}{\sum }}{\left(-1\right)}^{\frac{t\left(t-1\right)}{2}}{\langle x\rangle }_{t} \end{array}$$

The conjugation of multi-vector is denoted by


(12) $$\begin{array}{l} x^{*}=\overset{n}{\underset{t=0}{\sum }}{\left(-1\right)}^{\frac{t\left(t+1\right)}{2}}{\langle x\rangle }_{t} \end{array}$$

For any two multi-vectors *x, y*∈*G*_*n*_, dot product is defined by


(13) $$\begin{array}{l} x⊙y=\underset{i\in \Gamma }{\sum }{\left[x\right]}_{I}{\left[y\right]}_{I}e_{I} \end{array}$$

In addition, similar to quaternion, the basic element of GA also has the concept of module. For any multi-vector, its module is defined by


(14) $$\begin{array}{l} ||x||=\sqrt{\underset{I\in \Gamma }{\sum }{\left({\left[x\right]}_{I}\right)}^{2}} \end{array}$$

## 3. Methods

GA provides a new direction for the research and application of MTS. Through the latent structural dependencies within the multi-vector, the multi-dimensional features are combined into a single entity as input for network processing, and the internal relationship between the sequence features is captured, so that the inherent structural information in the multi-dimensional features is well preserved.

In this section, we extend RNN and LSTM from the real-value domain to the GA domain. In our proposed networks, inputs, outputs and weights are represented by GA multi-vectors. The operations in each layer and the training algorithm will be introduced in the following.

### 3.1. Geometric algebra based recurrent network layer

**Figure 1:**
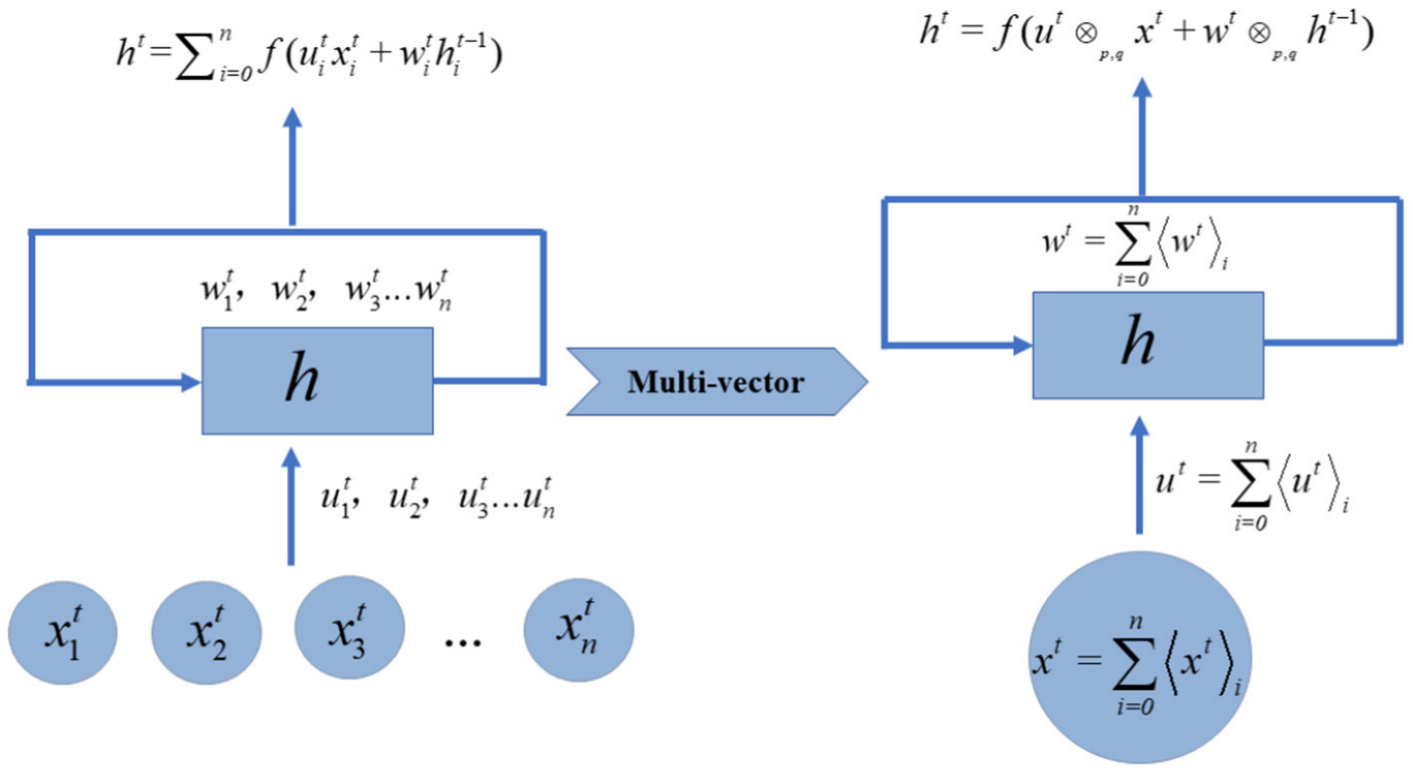
Multi-vectorization of real-valued RNN layer.

The learning process of the geometric algebra based RNN layer (GA-RNN) is similar to that of the real-valued RNN, the difference is that the input and network parameters have become multi-vectors, as shown in Figure 1. The multi-dimensional features at each time point are converted into multi-vectors as input for GA-RNN. The weights of the input features are also multi-vectors.

Suppose that the dimension of input vector *x*_*t*_ at time *t* is *N*, the number of neurons in the hidden layer is *H* and the number of neurons in output layer is *K*. In addition, σ and α represent the sigmoid and tanh activation functions, respectively. Then the forward propagation process of the GA-RNN basic unit is denoted by


(15) $$\begin{array}{l} \begin{array}{c} a^{t}=\sigma \left(U{⊗}_{p,q}x^{t}+W{⊗}_{p,q}a^{t-1}+\theta^{a}\right)\\ b^{t}=\alpha \left(V{⊗}_{p,q}a^{t}+\theta^{b}\right)\\ a^{t}={\left[\begin{array}{ccccc} a_{1}^{t} & \ldots & a_{h}^{t} & \ldots & a_{H}^{t} \end{array}\right]}^{T}\\ a^{t}={\left[\begin{array}{ccccc} b_{1}^{t} & \ldots & b_{k}^{t} & \ldots & b_{K}^{t} \end{array}\right]}^{T} \end{array} \end{array}$$

Where *x*^*t*^ and ***a***^*t*^ are multi-vector formed from the original input real-valued data and hidden state, respectively. ***U***, ***W***, and ***V*** are the weight matrices of the input, hidden state and output, respectively. ***θ***^*a*^ and ***θ***^*b*^ are the bias terms of the hidden state and output layer, respectively. ***b***^*t*^ is the output target.

In addition, for a multi-vector *x*, assuming that *f* is any standard activation function, then


(16) $$\begin{array}{l} f\left(x\right)=\underset{I\in \Gamma }{\sum }f\left({\left[x\right]}_{I}\right)e_{I} \end{array}$$

The activation function of multi-vector output is essentially the activation function operation in the real domain for each component of the multi-vector.

### 3.2. Geometric algebra based back-propagation through time

The principle of back-propagation algorithm through time of GA-RNN (GA-BPTT) is the same as that of real-valued RNN. After the sample information is modeled by GA, it is transmitted to the output layer through the input layer to obtain the actual output, and then the loss function is used to calculate the error E between actual output and true labels. Then backpropagation corrects the weight matrix and bias parameters, respectively, until the error converges to a certain threshold.

Similar to the multi-vector activation function, the loss function of multi-vector output is essentially the loss function in the real domain for each component. In the real domain, the dynamic loss is only based on all previously connected neurons, while the difference of GA-BPTT is that the multi-vector loss is calculated for each component of the multi-vector neuron parameter, which can act as a regularizer during training.

According to Equation (15), the output $b_{k}^{t}$ can be written as


(17) $$\begin{array}{l} b_{k}^{t}=\underset{I\in \Gamma }{\sum }{\left[b_{k}^{t}\right]}_{I}e_{I} \end{array}$$

Suppose that *y*^*t*^ is the true labels, *b*^*t*^ in Equation (15) is the actual output, the final loss function is defined as the sum of the mean squared errors at each moment


(18) $$\begin{array}{l} E=\overset{T}{\underset{t=1}{\sum }}E^{t}=\frac{1}{2}\overset{T}{\underset{t=1}{\sum }}||y^{t}-b^{t}|{|}_{2} \end{array}$$

Because the loss function is computed separately for each component of the multi-vector, the loss *E* is also a multi-vector.

#### 3.2.1. GA-RNN output layer weight matrix

The weight matrix ***V*** of the output layer is used to calculate the actual output *b*^*t*^, that is,


(19) $$\begin{array}{l} \begin{array}{c} \frac{\partial E}{\partial V}=\overset{T}{\underset{t=1}{\sum }}\frac{\partial E^{t}}{\partial V}\\ \frac{\partial E^{t}}{\partial V}=\left(\begin{array}{ccc} \frac{\partial E^{t}}{\partial v_{11}} & \cdots & \frac{\partial E^{t}}{\partial v_{H1}}\\ \vdots & \ddots & \vdots \\ \frac{\partial E^{t}}{\partial v_{1K}} & \cdots & \frac{\partial E^{t}}{\partial v_{HK}} \end{array}\right)=\left(\begin{array}{ccc} \nabla E_{v_{11}}^{t} & \ldots & \nabla E_{v_{H1}}^{t}\\ \vdots & \ddots & \vdots \\ \nabla E_{v_{1K}}^{t} & \cdots & \nabla E_{v_{HK}}^{t} \end{array}\right) \end{array} \end{array}$$

Each item in Equation (19) can be calculated individually, i.e.,


(20) $$\begin{array}{l} \nabla E_{v_{hk}}^{t}=\underset{I\in \Gamma }{\sum }\frac{\partial E^{t}}{\partial {\left[v_{hk}\right]}_{I}}e_{I} \end{array}$$

For each component in Equation (20), the chain rule is applied for parameter updating, that is,


(21) $$\begin{array}{l} \frac{\partial E^{t}}{\partial {\left(v_{hk}\right)}_{I}}=\underset{B\in \Gamma }{\sum }\frac{\partial E^{t}}{\partial {\left[m_{k}^{t}\right]}_{B}}\cdot \frac{\partial {\left[m_{k}^{t}\right]}_{B}}{\partial {\left[v_{hk}\right]}_{I}} \end{array}$$

The calculation of Equation (21) can be divided into the activation function part and the propagation function part, as shown in Equations (22, 23), respectively.


(22) $$\begin{array}{ll} \frac{\partial E^{t}}{\partial {\left[m_{k}^{t}\right]}_{B}}=\frac{\partial E^{t}}{\partial {\left[b_{k}^{t}\right]}_{B}}\cdot \frac{\partial {\left[b_{k}^{t}\right]}_{B}}{\partial {\left[m_{k}^{t}\right]}_{B}}=\left({\left[b_{k}^{t}\right]}_{B}-{\left[y_{k}^{t}\right]}_{B}\right)\cdot \alpha^{\prime }\left({\left[m_{k}^{t}\right]}_{B}\right)\\ = & {\left[\delta_{k}^{t}\right]}_{B} \end{array}$$

(23) $$\begin{array}{l} \begin{array}{c} \frac{\partial {\left[m_{k}^{t}\right]}_{B}}{\partial {\left[v_{hk}\right]}_{I}}={\left\{R\left(a_{h}^{t}\right)\right\}}_{\left(b,i\right)}\\ \frac{\partial {\left[m_{k}^{t}\right]}_{B}}{\partial {\left[a_{h}^{t}\right]}_{B}}={\left[v_{hk}\right]}_{0} \end{array} \end{array}$$

Where ${\left\{R\left(a_{h}^{t}\right)\right\}}_{\left(b,i\right)}$ is the value in row *b* column *i* of real representation matrix $R\left(a_{h}^{t}\right)$. Therefore,


(24) $$\begin{array}{l} \frac{\partial E^{t}}{\partial {\left[v_{hk}\right]}_{I}}=\underset{B\in \Gamma }{\sum }{\left[\delta_{k}^{t}\right]}_{B}\cdot {\left\{R\left(a_{h}^{t}\right)\right\}}_{\left(b,i\right)} \end{array}$$

That is,


(25) $$\begin{array}{l} \nabla E_{v_{jk}}^{t}=\underset{I\in \Gamma }{\sum }{\left[\underset{B\in \Gamma }{\sum }{\left[\delta_{k}^{t}\right]}_{B}\cdot {\left\{R\left(a_{h}^{t}\right)\right\}}_{\left(b,i\right)}\right]}_{I}e_{I} \end{array}$$

#### 3.2.2. GA-RNN hidden layer weight matrix

The derivation process of the backpropagation for ***W*** is as follows:


(26) $$\begin{array}{l} \frac{\partial E}{\partial W}=\overset{T}{\underset{t=1}{\sum }}\frac{\partial E^{t}}{\partial W} \end{array}$$

Similarly,


(27) $$\begin{array}{l} \frac{\partial E^{t}}{\partial W}=\left(\begin{array}{ccc} \frac{\partial E^{t}}{\partial w_{11}} & \cdots & \frac{\partial E^{t}}{\partial w_{H1}}\\ \vdots & \ddots & \vdots \\ \frac{\partial E^{t}}{\partial w_{1H}} & \cdots & \frac{\partial E^{t}}{\partial w_{HH}} \end{array}\right)=\left(\begin{array}{ccc} \nabla E_{w_{11}}^{t} & \cdots & \nabla E_{w_{H1}}^{t}\\ \vdots & \ddots & \vdots \\ \nabla E_{w_{1H}}^{t} & \cdots & \nabla E_{w_{HH}}^{t} \end{array}\right) \end{array}$$

Since the weights of the hidden layer are related to the state of the previous moment, consider


(28) $$\begin{array}{l} \begin{array}{c} a_{h}^{t+1}=\sigma \left(\overset{N}{\underset{i=1}{\sum }}u_{ih}{⊗}_{p,q}x_{i}^{t+1}+\overset{H}{\underset{h^{\prime }=1}{\sum }}w_{h^{\prime }h}{⊗}_{p,q}a_{h}^{t}+\theta_{h}^{a}\right)\\ b_{k}^{t+1}=\alpha \left(m_{k}^{t+1}\right)=\alpha \left(\overset{H}{\underset{h=1}{\sum }}v_{hk}{⊗}_{p,q}a_{h}^{t+1}+\theta_{k}^{b}\right) \end{array} \end{array}$$

We have


(29) $$\begin{array}{l} \nabla E_{w_{h^{\prime }h}}^{t}=\underset{I\in \Gamma }{\sum }\frac{\partial E^{t}}{\partial {\left[w_{h^{\prime }h}\right]}_{I}}e_{I} \end{array}$$

In Equation (29), the calculation of each component is denoted by


(30) $$\begin{array}{l} \begin{array}{c} \frac{\partial E^{t}}{\partial {\left[w_{h^{\prime }h}\right]}_{I}}=\underset{B\in \Gamma }{\sum }\frac{\partial E^{t}}{\partial {\left[m_{k}^{t}\right]}_{B}}\cdot \frac{\partial {\left[m_{k}^{t}\right]}_{B}}{\partial {\left[a_{h}^{t}\right]}_{B}}\cdot \frac{\partial {\left[a_{h}^{t}\right]}_{B}}{\partial {\left[z_{h}^{t}\right]}_{B}}\cdot \frac{\partial {\left[z_{h}^{t}\right]}_{B}}{\partial {\left[w_{h^{\prime }h}\right]}_{I}}\\ +\underset{B\in \Gamma }{\sum }\frac{\partial E^{t}}{\partial {\left[m_{k}^{t+1}\right]}_{B}}\cdot \frac{\partial {\left[m_{k}^{t+1}\right]}_{B}}{\partial {\left[a_{h}^{t+1}\right]}_{B}}\cdot \frac{\partial {\left[a_{h}^{t+1}\right]}_{B}}{\partial {\left[z_{h}^{t+1}\right]}_{B}}\cdot \frac{\partial {\left[z_{h}^{t+1}\right]}_{B}}{\partial {\left[w_{h^{\prime }h}\right]}_{I}} \end{array} \end{array}$$

Where


(31) $$\begin{array}{l} \begin{array}{cc} \underset{B\in \Gamma }{\sum }\frac{\partial E^{t}}{\partial {\left[m_{k}^{t}\right]}_{B}}\cdot \frac{\partial {\left[m_{k}^{t}\right]}_{B}}{\partial {\left[a_{h}^{t}\right]}_{B}}\cdot \frac{\partial {\left[a_{h}^{t}\right]}_{B}}{\partial {\left[z_{h}^{t}\right]}_{B}}\cdot \frac{\partial {\left[z_{h}^{t}\right]}_{B}}{\partial {\left[w_{h^{\prime }h}\right]}_{I}}\\ =\underset{B\in \Gamma }{\sum }{\left[\delta_{k}^{t}\right]}_{B}\cdot {\left[v_{hk}\right]}_{0}\cdot \sigma^{\prime }\left({\left[z_{h}^{t}\right]}_{B}\right)\cdot {\left\{R\left(a_{h}^{t-1}\right)\right\}}_{\left(b,i\right)}\\ + & \underset{B\in \Gamma }{\sum }\frac{\partial E^{t}}{\partial {\left[m_{k}^{t+1}\right]}_{B}}\cdot \frac{\partial {\left[m_{k}^{t+1}\right]}_{B}}{\partial {\left[a_{h}^{t+1}\right]}_{B}}\cdot \frac{\partial {\left[a_{h}^{t+1}\right]}_{B}}{\partial {\left[z_{h}^{t+1}\right]}_{B}}\cdot \frac{\partial {\left[z_{h}^{t+1}\right]}_{B}}{\partial {\left[w_{h^{\prime }h}\right]}_{I}}\\ =\underset{B\in \Gamma }{\sum }{\left[\delta_{k}^{t+1}\right]}_{B}\cdot {\left[v_{hk}\right]}_{0}\cdot \sigma^{\prime }\left({\left[z_{h}^{t+1}\right]}_{B}\right)\cdot {\left\{R\left(a_{h}^{t}\right)\right\}}_{\left(b,i\right)} \end{array} \end{array}$$

#### 3.2.3. GA-RNN input layer weight matrix

The updating of input layer weight matrix ***U*** is the same as that of the hidden layer, that is,


(32) $$\begin{array}{l} \begin{array}{c} \frac{\partial E}{\partial U}=\overset{T}{\underset{t=1}{\sum }}\frac{\partial E^{t}}{\partial U}\\ \frac{\partial E^{t}}{\partial U}=\left(\begin{array}{ccc} \frac{\partial E^{t}}{\partial u_{11}} & \cdots & \frac{\partial E^{t}}{\partial u_{N1}}\\ \vdots & \ddots & \vdots \\ \frac{\partial E^{t}}{\partial u_{1H}} & \cdots & \frac{\partial E^{t}}{\partial u_{NH}} \end{array}\right)=\left(\begin{array}{ccc} \nabla E_{u_{11}}^{t} & \cdots & \nabla E_{u_{N1}}^{t}\\ \vdots & \ddots & \vdots \\ \nabla E_{u_{1H}}^{t} & \cdots & \nabla E_{u_{NH}}^{t} \end{array}\right) \end{array} \end{array}$$

Where


(33) $$\begin{array}{l} \begin{array}{c} \nabla E_{u_{ih}}^{t}=\underset{I\in \Gamma }{\sum }\frac{\partial E^{t}}{\partial {\left[u_{ih}\right]}_{I}}e_{I}\\ \frac{\partial E^{t}}{\partial {\left(u_{ih}\right)}_{I}}=\underset{B\in \Gamma }{\sum }\frac{\partial E^{t}}{\partial {\left[m_{k}^{t}\right]}_{B}}\cdot \frac{\partial {\left[m_{k}^{t}\right]}_{B}}{\partial {\left[a_{h}^{t}\right]}_{B}}\cdot \frac{\partial {\left[a_{h}^{t}\right]}_{B}}{\partial {\left[z_{h}^{t}\right]}_{B}}\cdot \frac{\partial {\left[z_{h}^{t}\right]}_{B}}{\partial {\left[u_{ih}\right]}_{I}}\\ =\underset{B\in \Gamma }{\sum }{\left[\delta_{k}^{t}\right]}_{B}\cdot {\left(v_{hk}\right)}_{0}\cdot \sigma^{\prime }\left({\left[z_{h}^{t}\right]}_{B}\right)\cdot {\left\{R\left(x_{i}^{t}\right)\right\}}_{\left(b,i\right)} \end{array} \end{array}$$

That is,


(34) $$\begin{array}{l} \nabla E_{u_{ih}}^{t}=\underset{I\in \Gamma }{\sum }{\left[\underset{B\in \Gamma }{\sum }{\left[\delta_{h}^{t}\right]}_{B}\cdot {\left[v_{hk}\right]}_{0}\cdot \sigma^{\prime }\left({\left[z_{h}^{t}\right]}_{B}\right)\cdot {\left\{R\left(x_{i}^{t}\right)\right\}}_{\left(b,i\right)}\right]}_{I}e_{I} \end{array}$$

#### 3.2.4. GA-RNN output layer bias

$\frac{\partial E}{\partial \theta^{b}}$ can be written as


(35) $$\begin{array}{l} \frac{\partial E}{\partial \theta^{b}}=\overset{T}{\underset{t=1}{\sum }}\frac{\partial E^{t}}{\partial \theta^{b}}=\overset{T}{\underset{t=1}{\sum }}\left(\begin{array}{c} \frac{\partial E^{t}}{\partial \theta_{1}^{b}}\\ \vdots \\ \frac{\partial E^{t}}{\partial \theta_{K}^{b}} \end{array}\right)=\overset{T}{\underset{t=1}{\sum }}\left(\begin{array}{c} \nabla E_{\theta_{1}^{b}}^{t}\\ \vdots \\ \nabla E_{\theta_{K}^{b}}^{t} \end{array}\right) \end{array}$$

Similarly,


(36) $$\begin{array}{l} \begin{array}{c} \nabla E_{\theta_{k}^{b}}^{t}=\underset{I\in \Gamma }{\sum }\frac{\partial E^{t}}{\partial {\left[\theta_{k}^{b}\right]}_{I}}e_{I}\\ \frac{\partial E^{t}}{\partial {\left[\theta_{k}^{b}\right]}_{I}}=\underset{B\in \Gamma }{\sum }\frac{\partial E^{t}}{\partial {\left[m_{k}^{t}\right]}_{B}}\cdot \frac{\partial {\left[m_{k}^{t}\right]}_{B}}{\partial {\left[\theta_{k}^{b}\right]}_{I}}\\ =\underset{B\in \Gamma }{\sum }{\left[\delta_{k}^{t}\right]}_{B}\cdot \frac{\partial {\left[m_{k}^{t}\right]}_{B}}{\partial {\left[\theta_{k}^{b}\right]}_{I}}={\left[\delta_{k}^{t}\right]}_{I} \end{array} \end{array}$$

Therefore,


(37) $$\begin{array}{l} \nabla E_{\theta_{k}^{b}}^{t}=\underset{I\in \Gamma }{\sum }{\left[\delta_{k}^{t}\right]}_{I}e_{I} \end{array}$$

#### 3.2.5. GA-RNN hidden layer bias

Same as Equations (35, 36):


(38) $$\begin{array}{l} \nabla E_{\theta_{h}^{a}}^{t}=\underset{I\in \Gamma }{\sum }\frac{\partial E^{t}}{\partial {\left[\theta_{h}^{a}\right]}_{I}}e_{I} \end{array}$$

The difference is that the weight updating of the hidden layer takes into account the state of the previous moment, namely:


(39) $$\begin{array}{l} \begin{array}{c} \frac{\partial E^{t}}{\partial {\left[\theta_{h}^{a}\right]}_{I}}=\underset{B\in \Gamma }{\sum }\frac{\partial E^{t}}{\partial {\left[m_{k}^{t}\right]}_{B}}\cdot \frac{\partial {\left[m_{k}^{t}\right]}_{B}}{\partial {\left[a_{h}^{t}\right]}_{B}}\cdot \frac{\partial {\left[a_{h}^{t}\right]}_{B}}{\partial {\left[z_{h}^{t}\right]}_{B}}\cdot \frac{\partial {\left[z_{h}^{t}\right]}_{B}}{\partial {\left[\theta_{h}^{a}\right]}_{I}}\\ =\underset{B\in \Gamma }{\sum }{\left[\delta_{k}^{t}\right]}_{B}\cdot {\left[v_{hk}\right]}_{0}\cdot \sigma^{\prime }\left({\left[z_{h}^{t}\right]}_{B}\right)\cdot \frac{\partial {\left[z_{h}^{t}\right]}_{B}}{\partial {\left[\theta_{h}^{a}\right]}_{I}}\\ ={\left[\delta_{k}^{t}\right]}_{I}\cdot {\left[v_{hk}\right]}_{0}\cdot \sigma^{\prime }\left({\left[z_{h}^{t}\right]}_{I}\right) \end{array} \end{array}$$

Therefore,


(40) $$\begin{array}{l} \nabla E_{\theta_{h}^{a}}^{t}=\underset{I\in \Gamma }{\sum }{\left[\delta_{k}^{t}\right]}_{I}\cdot {\left[v_{hk}\right]}_{0}\cdot \sigma^{\prime }\left({\left[z_{h}^{t}\right]}_{I}\right)e_{I} \end{array}$$

### 3.3. Geometric algebra based long short-term memory network layer

Due to the “long-term dependence” problem caused by the disappearance of the RNN gradient, LSTM emerged. It has the ability to learn long-distance dependencies. This mechanism can be easily extended to the GA (GA-LSTM). Gates of LSTM is the core components, and the GA gates are characterized by the fusion process of each component of the multi-vector input signal after multiplication with the components of the multi-vector gate parameters. Let ***f***^*t*^, ***i***^*t*^, ***o***^*t*^, ***c***^*t*^, and ***h***^*t*^ be the forget gate, input gate, output gate, cell state and hidden state of the GA-LSTM unit at time step *t*, respectively. Then the GA-LSTM propagation process can be defined as:


(41) $$\begin{array}{l} \begin{array}{c} f^{t}=\sigma \left(U_{f}{⊗}_{p,q}x^{t}+W_{f}{⊗}_{p,q}h^{t-1}+\theta^{f}\right)\\ i^{t}=\sigma \left(U_{i}{⊗}_{p,q}x^{t}+W_{i}{⊗}_{p,q}h^{t-1}+\theta^{i}\right)\\ c^{t}=f^{t}⊙c^{t-1}+i^{t}⊙\alpha \left(U_{c}{⊗}_{p,q}x^{t}+W_{c}{⊗}_{p,q}h^{t-1}+\theta^{c}\right)\\ o^{t}=\sigma \left(U_{o}{⊗}_{p,q}x^{t}+W_{o}{⊗}_{p,q}h^{t-1}+\theta^{o}\right)\\ h^{t}=o^{t}⊙\alpha \left(c^{t}\right) \end{array} \end{array}$$

## 4. Results and discussion

### 4.1. Datasets

This experiment uses four publicly available datasets, namely Traffic, Electricity, Solar-Energy and Exchange-Rate. As shown in Table 1, Traffic records the occupancy rates (0–1) measured by simultaneous interpreting 862 different sensors on the San Francisco Bay expressway within 2 years (2015–2016 years), and the data are collected once 1 h. Electricity records the power consumption of 321 customers from 2012 to 2014. The data is collected every 15 min. This part converts the data to reflect the hourly consumption; Solar-Energy is the solar power generation record of 137 photovoltaic power stations in Alabama in 2006, which is sampled every 10 min. Exchange-Rate is the summary of daily exchange rates of eight countries including Australia, the United Kingdom, Canada, Switzerland, China, Japan, New Zealand and Singapore from 1990 to 2016. These datasets are real-world data and contain linear and nonlinear interdependencies (Jordan et al., 2003). All datasets are divided into training set (60%), verification set (20%) and test set (20%) in chronological order. The download address of the four datasets is here.

**Table 1:** Datasets used in the experiment.

**Dataset**	**Sequence length**	**Number of variables**	**Sampling interval**
Traffic	17,544	862	1 h
Electricity	26,304	321	1 h
Solar-Energy	52,560	173	10 min
Exchange-Rate	7,588	8	24 h

### 4.2. Experimental design

In order to verify the performance of the proposed neural networks based on GA, several MTS prediction algorithms with the proposed network are implemented, which are:

1) AR: Standard autoregressive model,

2) LSVR (Li and Cyrus, 2018): Vector autoregressive model with support vector regression objective function,

3) Lridge: Vector autoregressive model with L2 regularization,

4) TRMF (Hsiang et al., 2016): Autoregressive model of time regularization matrix decomposition,

5) GP (Roberts et al., 2012): Gaussian process for time-series modeling,

6) LSTNet (Lai et al., 2018): Deep neural network for modeling long-term and short-term time patterns,

7) GA-LSTNet: A geometric algebra based depth neural network for modeling long-term and short-term time patterns proposed in this paper.

For the first six comparison methods, the parameter setting used in this experiment is the same as that of Lai et al. (2018). That is, grid search is performed for all adjustable hyperparameters on the verification set of each method and each dataset. Specifically, the regularization coefficient of AR is selected from {0.1, 1, 10} to achieve the best performance. The value range of LSVR and LRidge regularization coefficients is {2^−10^, 2^−9^, …, 2^9^, 2^10^}. TRMF, the search ranges of hidden dimension and regularization coefficient are {2^2^, 2^3^, …, 2^6^} and {0.1, 1, 10}, respectively.

The GA-LSTNet used in this paper is composed of convolution layer and LSTM of GA instead of convolution layer and LSTM of LSTNet. LSTNet and GA-LSTNet have the same network structure and parameter settings. Their differences are the form of data input and the calculation method of network layer. Specifically, the selection range of hidden dimensions of LSTM and convolution layer is {50, 100, 200} and {20, 50, 100}. For the number of RNN-skip layers, Electricity and Traffic are set to 24, and the adjustment range of Solar-Energy and Exchange-Rate is 2^1^–2^6^. In addition to the input and output layers, dropout with a size of 0.1 or 0.2 is set after each layer. The optimizer for the two models is Adam.

In order to quantify and represent all experimental results, the seven methods in this section follow the same evaluation index (Hsiang et al., 2016): relative absolute error (RAE), root relative square error (RSE) and correlation coefficient (CORR). The first evaluation criterion RAE is defined as:


(42) $$\begin{array}{c} RAE=\frac{\sqrt{\sum_{t=t_{0}}^{t_{1}}\sum_{i=1}^{n}|\left(y_{t,i}-{ŷ}_{t,i}\right)|}}{\sqrt{\sum_{t=t_{0}}^{t_{1}}\sum_{i=1}^{n}|{ŷ}_{t,i}-\bar{{ŷ}_{t_{0}:t_{1},1:n}}|}} \end{array}$$

The second evaluation criterion RSE is defined as:


(43) $$\begin{array}{c} \mathrm{RSE}=\frac{\sqrt{\sum_{t=t_{0}}^{t_{1}}\sum_{i=1}^{n}{\left(y_{t,i}-{ŷ}_{t,i}\right)}^{2}}}{\sqrt{\sum_{t=t_{0}}^{t_{1}}\sum_{i=1}^{n}{\left({ŷ}_{t,i}-\bar{{ŷ}_{t_{0}:t_{1},1:n}}\right)}^{2}}} \end{array}$$

The third evaluation criterion CORR is defined as:


(44) $$\begin{array}{c} \mathrm{CORR}=\frac{1}{n}\sum_{i=1}^{n}\frac{\sum_{t=t_{0}}^{t_{1}}\left(y_{t,i}-\bar{y_{t_{0}:t_{1},i}}\right)\left({ŷ}_{t,i}-\bar{{ŷ}_{t_{0}:t_{1},i}}\right)}{\sqrt{\sum_{t=t_{0}}^{t_{1}}{\left(y_{t,i}-\bar{y_{t_{0}:t_{1},i}}\right)}^{2}{\left({ŷ}_{t,i}-\bar{{ŷ}_{t_{0}:t_{1},i}}\right)}^{2}}} \end{array}$$

Where *y* is the predicted value, ŷ is the real value of the test set, $\bar{y}$ represents the mean value of the set, and *t*∈[*t*_0_, *t*_1_] is the data label of the test set. For RAE and RSE, the lower the value, the better the prediction result. On the contrary, for CORR, the higher the value, the better the prediction result.

### 4.3. Experimental results and analysis

In this part, seven methods will be used to conduct prediction experiments on four datasets, and the prediction range horizon is set to {3, 6, 12, 24}. According to Table 1, for Electricity and Traffic, the prediction range is set to {3, 6, 12, 24} h. For Solar-Energy, the prediction range is set to {30, 60, 120, 240} min. The prediction range for Exchange-Rate is set to {3, 6, 12, 24} days.

**Figure 2:**
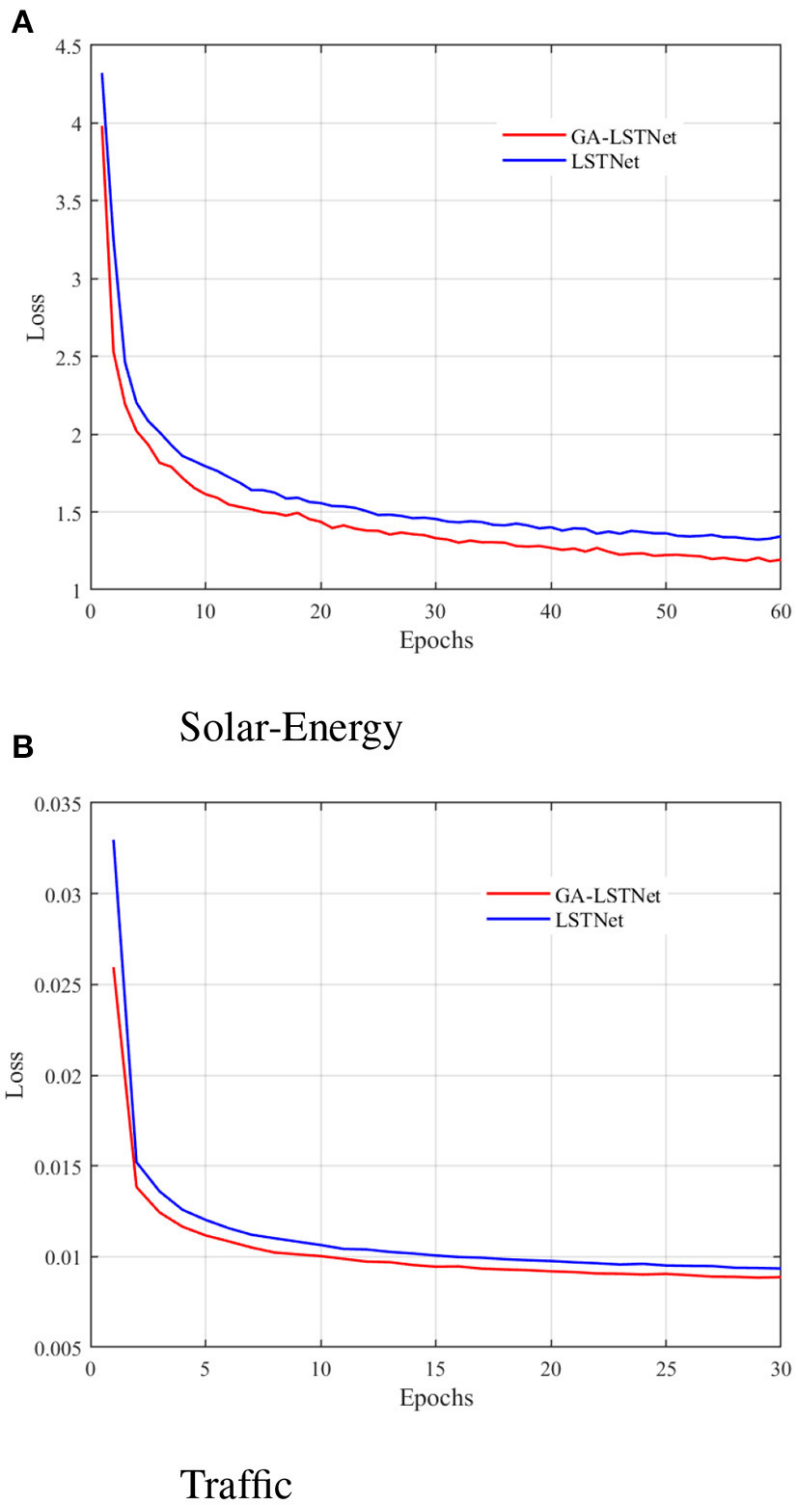
Convergence curves of GA-LSTNet and LSTNet on different datasets. **(A)** Solar-Energy and **(B)** Traffic.

This part first compares the convergence curves of the two networks on Traffic and Solar-Energy. Figure 2A shows the convergence curve of GA-LSTNet and LSTNet predicting power generation in the next 240 min on the Solar-Energy dataset; Figure 2B shows the convergence curve of GA-LSTNet and LSTNet predicting traffic flow in the next 6 h on the Traffic dataset. The curves of the two experiments show that under the same network configuration, the convergence rate of GA-LSTNet is faster than LSTNet, and the final convergence value and the training error are smaller.

Tables 2, 3, respectively, show the comparison of RAE and RSE of the prediction results of the seven methods, in which the best results are shown in bold.

**Table 2:** Prediction results using RAE as indicator.

**Dataset**	**Horizon**	**AR**	**LSVR**	**LRidge**	**TRMF**	**GP**	**LSTNet**	**GA-LSTNet**
Traffic	3	0.4491	0.4929	0.4965	0.5823	0.5148	0.3238	**0.311**
	6	0.461	0.5483	0.5115	0.4653	0.5759	0.3254	**0.3144**
	12	0.47	0.7454	0.5198	0.4868	0.5316	0.3433	**0.3308**
	24	0.4696	0.4761	0.4846	0.5352	0.4829	0.354	**0.3357**
Electricity	3	0.0579	0.0858	0.09	0.1032	0.0907	0.0493	**0.0485**
	6	0.0598	0.0816	0.0933	0.1269	0.1137	**0.0526**	0.0541
	12	0.0603	0.0762	0.1268	0.1328	0.1043	**0.0534**	0.0561
	24	0.0611	0.069	0.0779	0.201	0.0776	**0.0545**	0.0593
Solar-Energy	3	0.1846	**0.1082**	0.1227	0.1326	0.1419	0.1462	0.1083
	6	0.3242	0.2451	0.2098	0.1984	0.2189	0.1462	**0.1315**
	12	0.5673	0.4362	0.407	0.4786	0.4095	**0.1917**	0.1959
	24	0.9221	0.618	0.6977	0.9527	0.7599	0.3042	**0.2542**
Exchange-Rate	3	0.0181	0.0148	**0.0144**	0.0289	0.023	0.0364	0.0189
	6	0.0224	0.0231	0.0225	0.0517	0.0239	**0.0207**	0.025
	12	0.0291	0.036	0.0358	0.0429	0.0355	**0.0286**	0.0323
	24	**0.0378**	0.0571	0.0602	0.058	0.0547	0.0475	0.0505

The bold values indicate the best RAE and RSE of the prediction results of the seven methods.

**Table 3:** Prediction results using RSE as indicator.

**Dataset**	**Horizon**	**AR**	**LSVR**	**LRidge**	**TRMF**	**GP**	**LSTNet**	**GA-LSTNet**
Traffic	3	0.5991	0.574	0.5833	0.6708	0.6082	0.4896	**0.4798**
	6	0.6218	0.658	0.592	0.6261	0.6772	0.4909	**0.4852**
	12	0.6252	0.7714	0.6148	0.5956	0.6406	0.507	**0.5002**
	24	0.6293	0.5909	0.6025	0.6442	0.5995	0.5188	**0.5026**
Electricity	3	0.0995	0.1523	0.1467	0.1802	0.15	0.0851	**0.0844**
	6	0.1035	0.1372	0.1419	0.2039	0.1907	0.0941	**0.0935**
	12	0.105	0.1333	0.2129	0.2189	0.1621	**0.0967**	0.0975
	24	0.1054	0.118	0.128	0.3656	0.1273	**0.1046**	0.1101
Solar-Energy	3	0.2435	0.2021	0.2019	0.2473	0.2259	0.2067	**0.1976**
	6	0.379	0.2999	0.2954	0.347	0.3286	0.2606	**0.2462**
	12	0.5911	0.4846	0.4832	0.5597	0.52	0.3563	**0.3445**
	24	0.8699	0.73	0.7287	0.9005	0.7973	0.4695	**0.4306**
Exchange-Rate	3	0.0228	0.0189	**0.0184**	0.0351	0.0239	0.0431	0.0234
	6	0.0279	0.0284	0.0274	0.0875	0.0272	**0.0256**	0.0297
	12	0.0353	0.0425	0.0419	0.0494	0.0394	**0.0352**	0.0401
	24	**0.0445**	0.0662	0.0675	0.0653	0.058	0.0475	0.0594

The bold values indicate the best RAE and RSE of the prediction results of the seven methods.

It can be seen from the results in Tables 2, 3 that on the Traffic and Solar-Energy datasets, the GA- LSTNet model proposed in this part has absolute advantages over the LSTNet model with the same structure and achieves lower prediction error. On the Electricity dataset, although LSTNet achieved the best results in some predictions, it can be observed that the difference between GA-LSTNet and LSTNet is no more than 0.002, which is a very small gap. The experimental results show the feasibility of GA-LSTNet, because it can capture more useful information in different data channels when it represents MTS as GA multi-vector. Therefore, the LSTM based on GA has better performance than the real-valued LSTM in MTS prediction.

**Figure 3:**
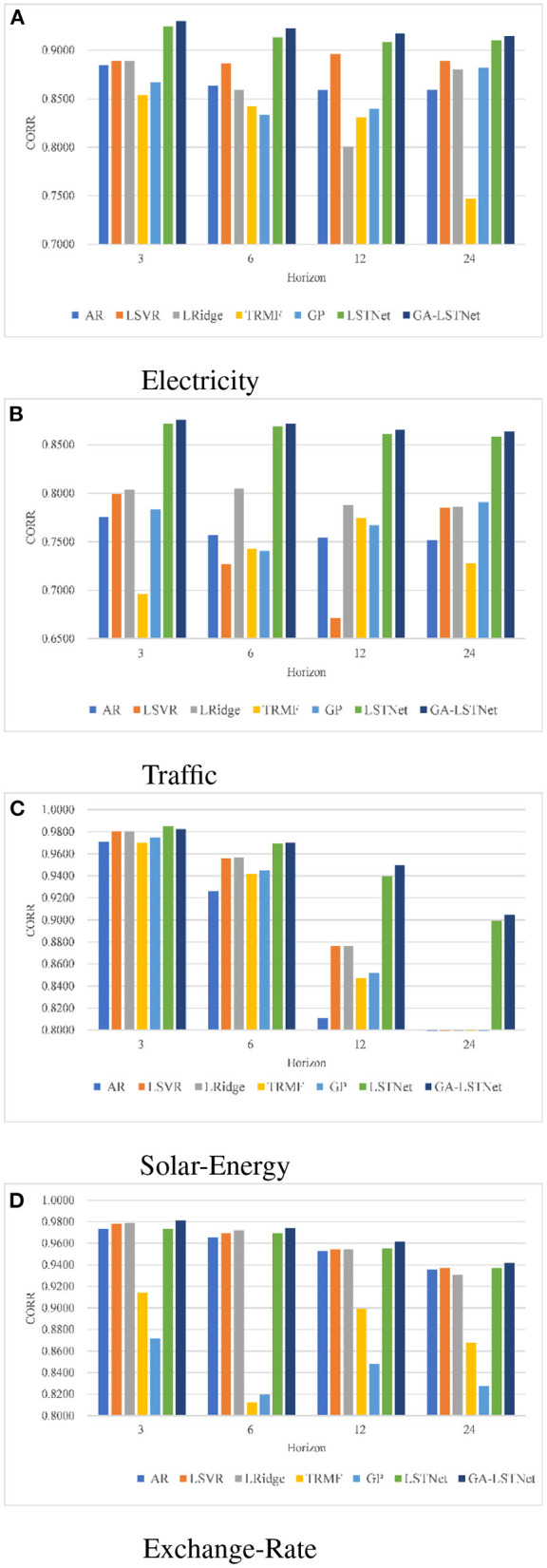
Prediction results using CORR. **(A)** Electricity, **(B)** Traffic, **(C)** Solar-Energy, and **(D)** Exchange-Rate.

In addition, in order to more intuitively observe the prediction results, Figure 3 shows the prediction results of seven different methods under different conditions using CORR as an index. In Figure 3, the higher the bar chart, the greater the CORR representing the corresponding prediction task and the higher the prediction accuracy. As shown in Figure 3, GA-LSTNet obtained the highest CORR value in each prediction task of Electricity, Traffic and Exchange-Rate. In the prediction task of *horizon* = 3 for Solar-Energy dataset, the accuracy is slightly lower than LSTNet, but with the growth of prediction time, the superiority of GA-LSTNet is gradually obvious. It can be seen that the introduction of GA into real-valued RNN and LSTM will not change their basic properties. The capture of the correlation between different features by GA enhances the long-term dependent learning ability of real-valued neural networks.

**Figure 4:**
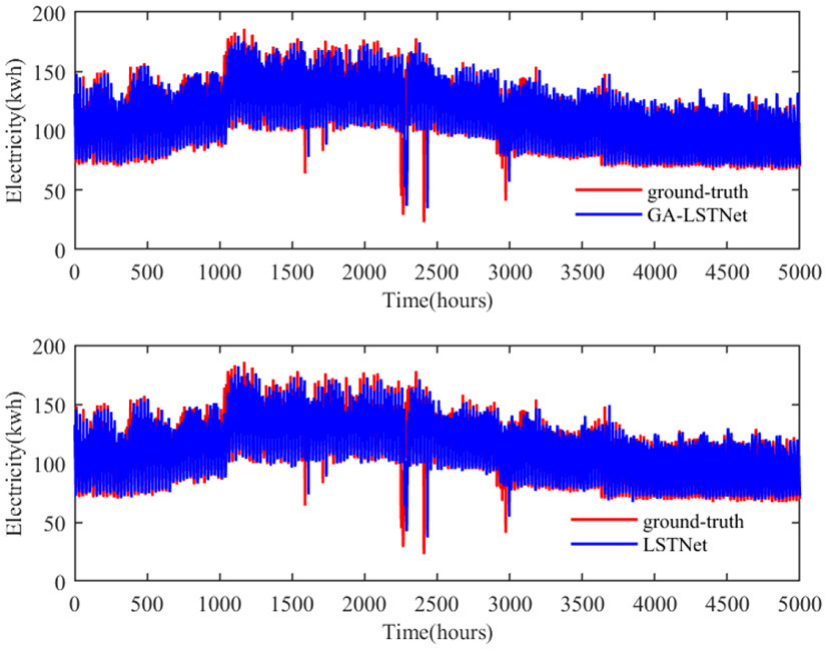
Prediction results of electricity by LSTNet and GA-LSTNet when *horizon* = 12.

**Figure 5:**
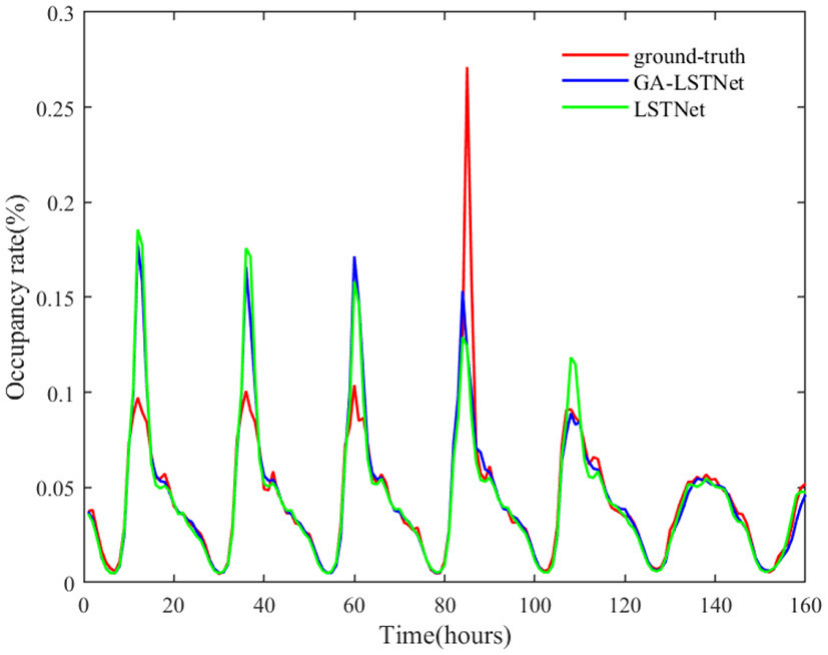
Prediction results of traffic by LSTNet and GA-LSTNet when *horizon* = 6.

From the above quantitative results, it can be seen that the overall accuracy of GA-LSTNet is improved compared with LSTNet, which means that the predicted positions of some points will be more consistent with the real labels. As shown in Figures 4, 5, two networks are selected to visually observe all the prediction results of Electricity and some prediction results on Traffic dataset.

In Figure 4, red represents the real value of power consumption and blue represents the predicted value. GA-LSTNet and LSTNet are similar to the real value trend on the whole, but GA-LSTNet performs better in details, especially in some prominent troughs. For example, in 2,000–2,500 h, the gap between GA-LSTNet and the real value is smaller and closer to the real value.

In Figure 5, the red curve represents the real value of Traffic occupancy within 160 h of the test set, the green curve represents the predicted value of LSTNet, and the blue curve represents the predicted value of GA-LSTNet. Both are basically consistent with the real value at the trough, but among the six peaks shown in Figure 5, five are GA-LSTNet, which is closer to the real value. It can be seen from the comparison figure that after replacing the corresponding real-valued network with GA convolution and LSTM, GA-LSTNet retains more useful relevant information in the original data due to the multi-dimensional consistency of GA, and the prediction performance is significantly improved.

## 5. Conclusion

This paper focuses on the construction of the geometric algebra based RNN and LSTM for the processing of MTS. Under the framework of GA, the MTS is encoded into GA multi-vectors to avoid the loss of structural relationships among multi-dimensional variables. And then the forward and backpropagation algorithms for the proposed GA-RNN, GA-LSTM are derived. The experimental results show that the GA-LSTNet has good convergence and more accurate prediction accuracy in MTS prediction, and has certain advantages compared with the real-valued LSTNet. GA-RNN and GA-LSTM provide a more accurate solution for the existing shortcomings of MTS prediction models. At next steps, we will focus on more practical MTS applications with the proposed networks.

## Data availability statement

The original contributions presented in the study are included in the article/supplementary material, further inquiries can be directed to the corresponding authors.

## Author contributions

YL and YueW proposed the new idea of the paper and participated in the outage performance analysis. YiW performed the simulations and drafted the paper. CQ played an important role in interpreting the results and revised the manuscript. RW conceived of the study, participated in its design, coordination, and helped to draft the manuscript. All authors have read and approved the final manuscript.
